# Integrating Network Pharmacology and Experimental Validation of Oleanolic Acid Targeting the PPARα-CPT1A Axis to Modulate Lipid Metabolism in Hepatocellular Carcinoma Cells

**DOI:** 10.3390/ijms27104595

**Published:** 2026-05-20

**Authors:** Yuxin Liu, Deru Zhang, Dan Liu, Mengke Wang, Hanning Lyu, Yang Sun

**Affiliations:** College of Basic Medicine, Heilongjiang University of Chinese Medicine, Harbin 150040, China; 18833575139@163.com (Y.L.); 13942698906@163.com (D.Z.); ld20190218@163.com (D.L.); wyyxwmk@163.com (M.W.); lhn6792024@163.com (H.L.)

**Keywords:** oleanolic acid, liver cancer, lipid metabolism, PPARα-CPT1A axis

## Abstract

Patients with liver cancer frequently exhibit abnormal liver function and disorders in lipid metabolism. This study investigates the effects of Oleanolic acid (OA) on hepatocellular carcinoma (HCC) through the regulation of lipid metabolism. Computational simulations identified six core targets of OA, including PPARα, HMGCR, and ESR1, with stable binding confirmed through molecular docking and dynamics analyses. The experiments demonstrated that OA reduced intracellular lipid accumulation, suppressed cell migration (*p* < 0.05), and promoted apoptosis. The levels of lipid droplets and triglycerides (TG) were significantly decreased (*p* < 0.05). The expression levels of lipid metabolism-related genes, including *PPARA*, *CPT1A*, *FASN*, and *HMGCR*, were assessed using qRT-PCR (*p* < 0.05). Additionally, protein expression levels were analyzed through Western blotting (*p* < 0.05). Furthermore, the combination of OA with the antagonist GW6471 enhanced tumor suppression, while the combination with the agonist Pemafibrate reversed the effects of OA. Compared to OA alone, the antagonist combination significantly reduced PPARα and CPT1A protein expression (*p* < 0.05), whereas Pemafibrate upregulated these proteins (*p* < 0.05). In conclusion, OA exerts its anti-lipid metabolism effects in HCC by modulating the PPARα-CPT1A axis, indicating its potential therapeutic value in liver cancer treatment.

## 1. Introduction

Hepatocellular Carcinoma (HCC) is a prevalent malignant tumor of the digestive system, ranking as the sixth-most common malignant tumor worldwide and the third leading cause of cancer-related mortality [1]. In recent years, metabolic dysfunction-associated steatotic liver disease (MASLD) has emerged as a significant etiological factor for HCC. Among patients with MASLD-related HCC, approximately 40% to 50% can progress directly to HCC without passing through the cirrhosis stage [2]. Within the HCC microenvironment, lipids not only provide energy for tumor cells through abnormal metabolism but also regulate multiple signaling pathways [3,4]. Cancer cells in the tumor microenvironment undergo lipid metabolic reprogramming, utilizing aberrant lipid metabolism at various stages to support rapid proliferation, deplete nutrients from the tumor microenvironment, and ultimately facilitate invasion and metastasis [5]. For the systemic treatment of advanced HCC, the primary approach involves the use of immune checkpoint inhibitors in combination with anti-angiogenic targeted therapies, such as atezolizumab plus bevacizumab [6]. However, most patients develop primary or secondary drug resistance, underscoring the clinical importance of exploring novel therapeutic targets based on metabolic regulation. Therefore, targeting lipid metabolism in HCC may represent a promising therapeutic strategy for liver cancer.

Oleanolic acid (OA) is a pentacyclic triterpenoid compound that is widely distributed in medicinal plants, including *Ligustrum lucidum*, *Prunella vulgaris*, and *Scutellaria baicalensis*. It has been shown to exhibit a range of biological activities, such as hepatoprotection, lipid-lowering effects, and antitumor properties [7]. Given its significant bioactivity, recent research has focused on optimizing the extraction and purification processes of OA, along with structural modifications intended to enhance its bioavailability [8]. In the realm of cancer treatment, OA demonstrates antitumor effects through various mechanisms, including the inhibition of tumor cell proliferation, suppression of tumor cell migration and invasion, and induction of tumor cell apoptosis [9,10]. Nevertheless, prior studies have not systematically explored the potential of OA to exert anti-HCC effects through the regulation of lipid metabolism. Recently, the integration of network pharmacology with molecular dynamics simulation has emerged as a powerful approach for elucidating the multi-target and multi-pathway synergistic mechanisms of natural products. However, the application of this strategy to investigate the role of OA in regulating lipid metabolism in liver cancer remains unreported.

Previous studies have demonstrated that OA significantly inhibits the proliferation of HCC SMMC-7721 cells [11]; however, the underlying mechanisms remain unclear. In this study, we employed network pharmacology to predict potential targets of OA in the treatment of liver cancer, complemented by molecular docking and dynamics simulations to validate key interaction sites. Through in vitro cell experiments and BALB/c mouse models, we aimed to systematically elucidate the molecular mechanisms by which OA exerts its anti-liver cancer effects by intervening in lipid metabolism.

## 2. Results

### 2.1. In Silico Analysis of Molecular Targets and Experimental Validation

#### 2.1.1. Network Pharmacology Analysis

##### Identification of OA- and Liver Cancer-Related Targets

**Figure 1:**
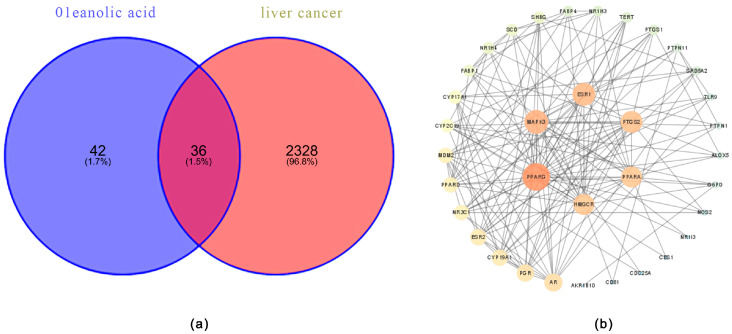
(**a**) Venn diagram showing the intersection of OA-related targets and liver cancer-related targets. The overlapping region represents the common targets shared by both diseases. (**b**) PPI network of the 56 intersected targets. Nodes represent target proteins, and edges indicate known interactions from the STRING database (confidence score ≥ 0.4). Node size is proportional to degree centrality.

A total of 78 targets for OA and 2364 targets related to liver cancer were identified through database searches. Among these, 36 intersecting targets were found, indicating that OA may exert therapeutic effects on liver cancer through these potential targets (Figure 1a).

##### Development of the Protein–Protein Interaction (PPI) Network and Drug–Component–Target Key Pathway Map

The PPI network was constructed using 36 intersecting genes, resulting in a network comprising 26 nodes and 161 edges. To further investigate the relationship between OA and liver cancer, a drug–component–target key pathway map was created for visualization. This analysis elucidated that OA influences liver cancer through multiple targets. Notably, six genes emerged as key potential targets for OA in the treatment of liver cancer: peroxisome proliferator-activated receptor gamma (PPARG), mitogen-activated protein kinase 3 (MAPK3), prostaglandin–endoperoxide synthase 2 (PTGS2), estrogen receptor 1 (ESR1), 3-hydroxy-3-methylglutaryl-CoA reductase (HMGCR), and peroxisome proliferator-activated receptor alpha (PPARA) (Figure 1b).

##### GO Enrichment Analysis and KEGG Enrichment Analysis

**Figure 2:**
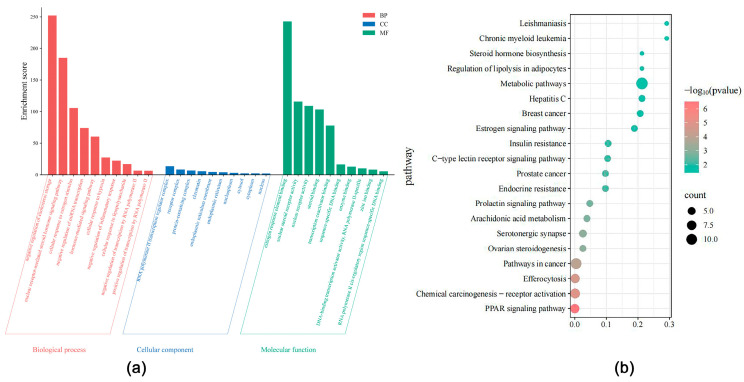
Enrichment Analysis. (**a**) GO enrichment analysis of the 56 OA-HCC common targets. The results are categorized into BP, CC and MF. (**b**) KEGG pathway enrichment analysis of the same 56 common targets. Bubble plots display the top 20 significantly enriched signaling pathways, where bubble size represents gene count and color indicates adjusted *p*-value.

The GO enrichment analysis identified a total of 114 biological processes (BP), 13 molecular functions (MF), and 55 cellular components (CC). Notable biological processes included cellular responses to hypoxia, cholesterol storage, and the negative regulation of inflammatory responses. Major cellular components encompassed cytoplasmic and nuclear structures, membranous organelles, and macromolecular complexes, while molecular functions primarily involved nuclear receptor activity and steroid binding. The top 10 terms from each GO category, ranked by *p*-value, were visualized. Additionally, the KEGG enrichment analysis revealed 20 pathways, with significant pathways including the PPAR signaling cascade, prolactin receptor pathway, and C-type lectin receptor signaling mechanism (Figure 2a,b).

#### 2.1.2. Molecular Docking

**Figure 3:**
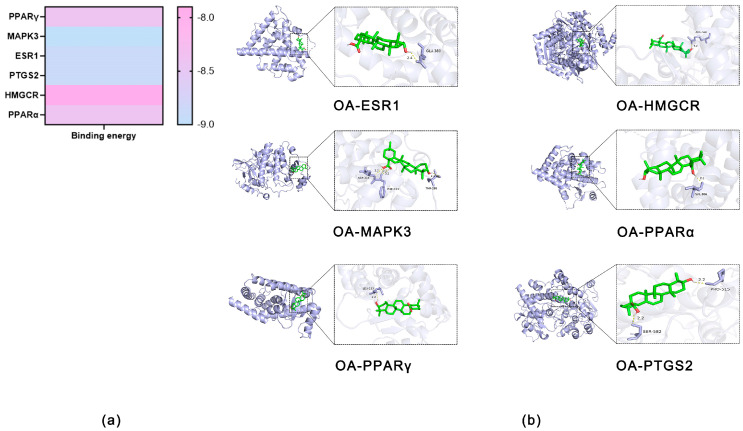
(**a**) Heatmap of molecular docking binding affinities (-kcal/mol) between candidate compounds OA and OA-HCC common target proteins. colder colors indicate stronger binding affinity. (**b**) 3D representation of the docking pose of OA bound to the active site of the target protein.

Molecular docking analyses were sequentially conducted between OA and six protein targets. It is well established that lower binding energy correlates with stronger binding affinity. The docking analyses revealed that the binding energies between OA and all six core targets were below −6 kcal/mol, indicating favorable binding interactions (Figure 3a,b and Table 1).

#### 2.1.3. Molecular Dynamics (MD) Simulations

To validate the dynamic stability of the ligand–receptor complex, 100 ns MD simulations were conducted to examine the binding conformations of OA with each target protein.

##### Analysis of Dynamical Stability and Conformational Changes

**Figure 4:**
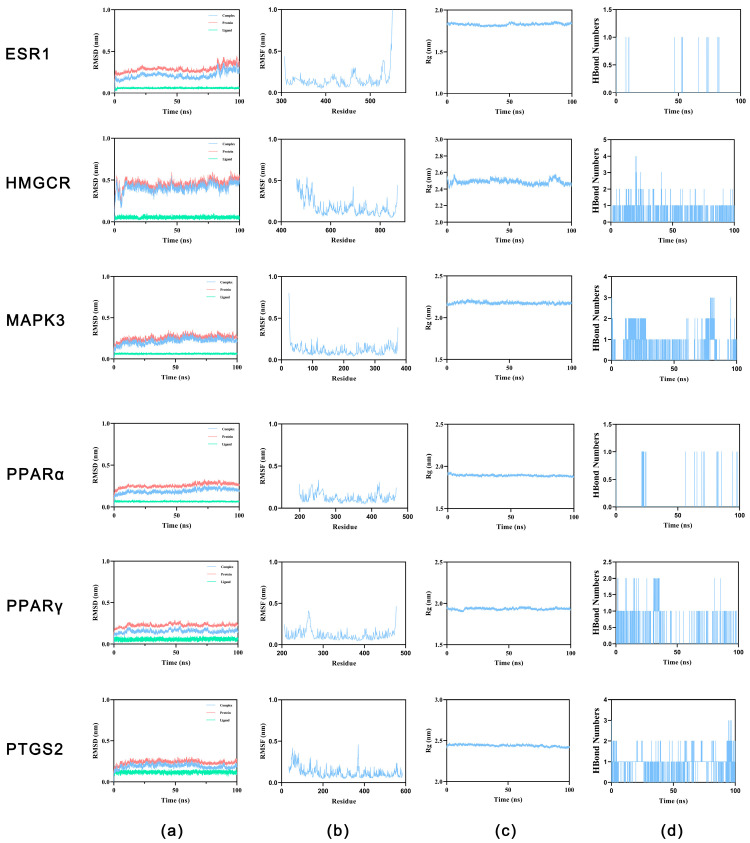
MD simulation analysis of OA bound to six target proteins. (**a**) RMSD of the protein backbone over 100 ns simulation time. (**b**) RMSF per residue, indicating flexibility of individual residues. (**c**) Rg, reflecting the compactness of each protein structure. (**d**) Number of HBonds formed between OA and each target protein over the simulation trajectory.

To evaluate the dynamic stability of the complex, we calculated the root-mean-square deviation (RMSD), root-mean-square fluctuation (RMSF), radius of gyration (Rg) and the number of hydrogen bonds. The ligand remained stable within the binding pocket, and the complexes maintained compact globular conformations throughout the 100 ns trajectory (Figure 4a–d).

##### Free Energy Landscape (FEL) Analysis

FELs were constructed using RMSD, Rg and Gibbs free energy data, employing a color scale that ranges from high energy (red) to low energy (blue). Both 3D and 2D FEL topology maps were generated for enhanced visualization.

**Figure 5:**
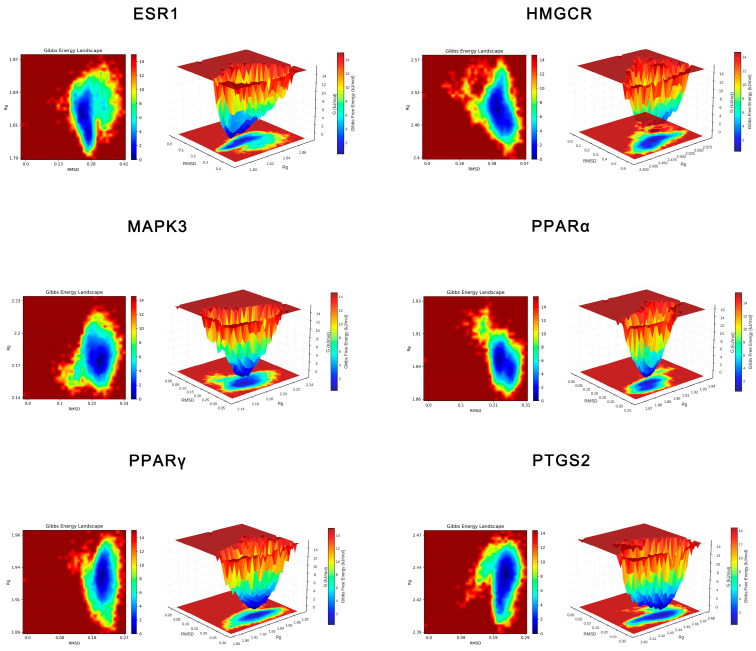
FEL analysis of the OA–target complex. Both 3D and 2D topology maps are presented for visualization.

The FEL color maps, with red indicating high energy and blue indicating low energy, revealed a deep-blue global minimum for OA–receptor complexes, suggesting the presence of compact structures. The stability of these complexes varied, with PPARγ, ESR1, PPARα and HMGCR exhibiting greater rigidity and compactness, warranting further investigation (Figure 5).

##### Molecular Mechanics Poisson–Boltzmann Surface Area (MM/PBSA)-Predicted Binding Free Energy and Energy Decomposition

The binding free energies for MM/PBSA were calculated based on the last 20 ns of stable MD trajectories, encompassing 100 frames. All complexes demonstrated negative binding free energies, indicating favorable interactions. Notably, ESR1, HMGCR, and PPARα exhibited binding free energy values below −25 kcal/mol, suggesting relatively higher affinities for OA (Table 2).

#### 2.1.4. Comprehensive Screening of Core Targets

Based on integrated molecular docking, MD simulations, and MM/PBSA calculations, six core targets—PPARγ, MAPK3, ESR1, PTGS2, HMGCR and PPARα—were evaluated. Among these, ESR1, HMGCR and PPARα demonstrated the highest binding affinities, with MM/PBSA energies of −31.84, −31.08 and −28.20 kcal/mol, respectively. All three targets exhibited minimal RMSD fluctuations (ESR1: 0.15–0.35 nm; HMGCR: 0.35–0.50 nm; PPARα: 0.15–0.25 nm), stable Rg curves and concentrated FEL energy basins, indicating stable dynamic binding. KEGG analysis revealed significant enrichment in the PPAR signaling and metabolic pathways. Both PPARα and HMGCR are implicated in lipid metabolism, which aligns with the enrichment results, leading to their selection as core targets for experimental validation.

### 2.2. Mechanism of OA in Regulating Lipid Metabolism

#### 2.2.1. Impact of Various Concentrations of OA on the Viability of Huh-7 Cells

**Figure 6:**
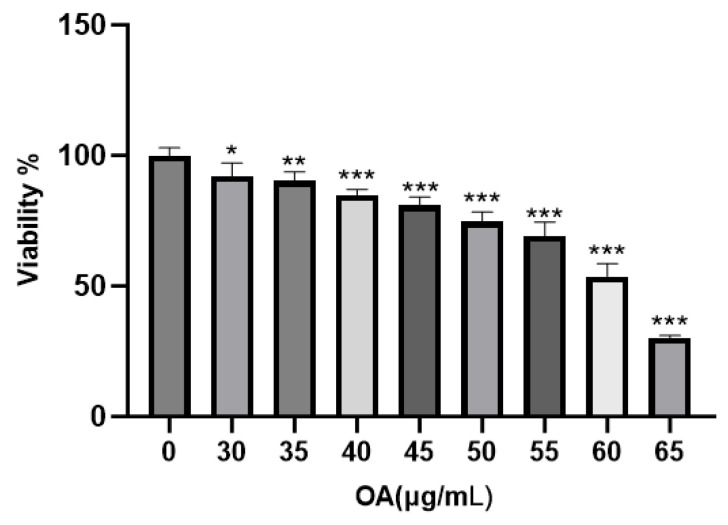
Effect of OA on the viability of Huh-7 cells. Cells were treated with OA at the indicated concentrations for 24 h. Data are presented as the mean ± SD (*n* = 4 independent replicates per group). * *p* < 0.05, ** *p* < 0.01, *** *p* < 0.001 vs. the Control group. The experiment was independently repeated three times.

OA significantly reduced cell viability at concentrations exceeding 30 μg/mL. Across a gradient of 30 to 65 μg/mL, OA inhibited cell viability in a concentration-dependent manner, with significant effects observed at concentrations of 40 μg/mL or higher (*p* < 0.01) compared to the Control group. The calculated IC50 value was 59.09 ± 2.58 μg/mL. Importantly, a marked decline in cell viability was noted at concentrations of 60 μg/mL and above, indicating potential cytotoxic effects within this range (Figure 6).

#### 2.2.2. Hoechst 33342/PI Staining Determines the Optimal Concentration of OA for Intervention

**Figure 7:**
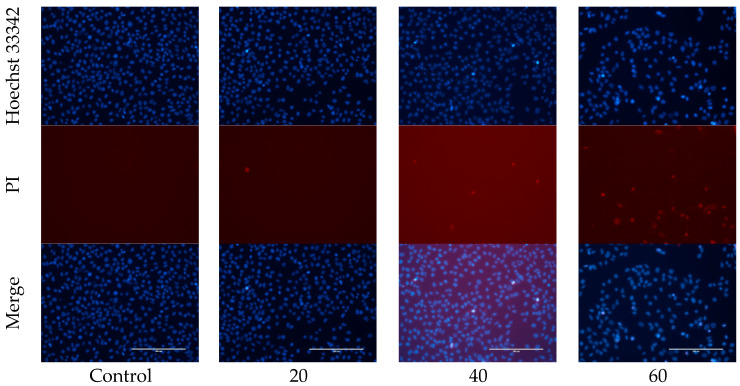
Representative images of Hoechst 33342/PI double staining in Huh-7 cells treated with Control, 20 μg/mL OA, 40 μg/mL OA and 60 μg/mL OA (Scale bar = 200 μm).

Microscopic examination of Huh-7 cells revealed concentration-dependent changes. At low concentrations (20 μg/mL), cell density was comparable to the Control group, with occasional blue fluorescence (indicative of apoptosis) and red fluorescence (indicative of necrosis). At medium concentrations (40 μg/mL), there was a moderate density of cells, with an increase in both apoptotic and necrotic cells. At high concentrations (60 μg/mL), cell density was significantly reduced, with extensive red fluorescence indicating widespread necrosis and cytotoxicity. The 40 μg/mL concentration, which effectively induced apoptosis while limiting necrosis, was selected as the optimal dose for subsequent experiments. To further evaluate the selectivity of OA, normal liver cells (THLE-2) were treated with the same concentration (40 μg/mL) for 24 h to observe any morphological changes. In comparison to Huh-7 cells, fewer THLE-2 cells underwent apoptosis (Figure S2). Additionally, observations under an inverted microscope revealed that the morphology of THLE-2 cells remained largely intact following OA treatment, with only a minor proportion of cells becoming rounded and detached (Figure S1). These results indicate that, at concentrations effective in inhibiting liver cancer cells, OA exhibits low toxicity to normal liver cells, demonstrating a degree of selectivity (Figure 7).

#### 2.2.3. Effect of OA on Lipid Accumulation in Huh-7 Cells

**Figure 8:**
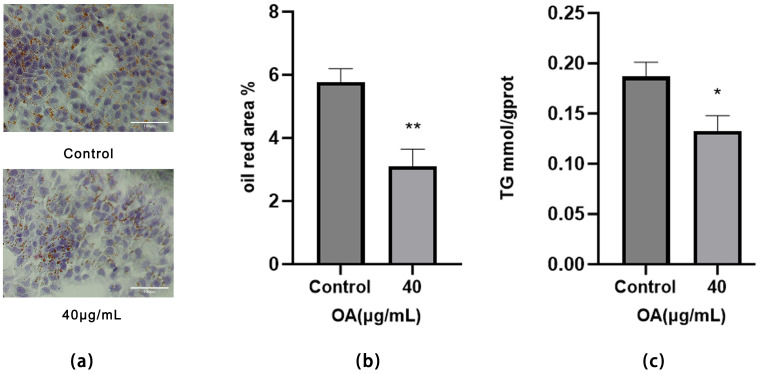
(**a**) Representative images of Oil Red O staining in Huh-7 cells from the Control and OA 40 μg/mL (Scale bar = 100 μm). (**b**) Statistical analysis results of Oil Red O-stained images. Cells were treated with OA at the indicated concentrations for 24 h. Data are presented as the mean ± SD (*n* = 3 independent replicates per group). ** *p* < 0.01 vs. the Control group. The experiment was independently repeated three times. (**c**) TG levels of Huh-7 cells. Cells were treated with OA at the indicated concentrations for 24 h. Data are presented as the mean ± SD (*n* = 3 independent replicates per group). * *p* < 0.05 vs. the Control group. The experiment was independently repeated three times.

Compared to the Control group, treatment with 40 μg/mL OA significantly reduced the number of lipid droplets in Huh-7 cells, decreased their diameter, and resulted in lighter red staining, indicating a reduction in lipid accumulation (*p* < 0.01) (Figure 8a,b). These results indicate that OA reduces lipid accumulation in HCC cells.

#### 2.2.4. Effect of OA on TG Levels in Huh-7 Cells

The experimental results indicate that, in comparison to the Control group, TG levels were significantly decreased after 24 h of OA treatment (*p* < 0.05) (Figure 8c). This result aligns with the Oil Red O staining findings, further indicating that OA attenuates lipid accumulation.

#### 2.2.5. Effect of OA on the Migration of Huh-7 Cells

**Figure 9:**
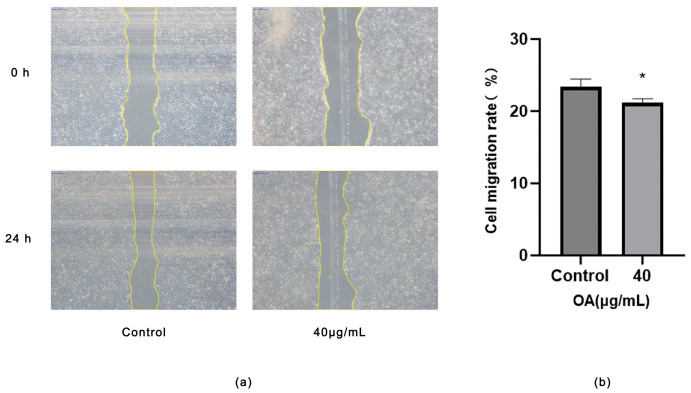
(**a**) Representative micrographs of the wound area at 0 h and 24 h after scratch wounding in Huh-7 cells. Cells were treated with Control or 40 μg/mL OA. (Scale bar = 315 μm). (**b**) Statistical analysis of cell migration rates. Cells were treated with OA at the indicated concentrations for 24 h. Data are presented as the mean ± SD (*n* = 3 independent replicates per group). * *p* < 0.05 vs. the Control group. The experiment was independently repeated three times.

Compared to the Control group, treatment with 40 μg/mL OA for 24 h significantly impaired scratch wound closure in Huh-7 cells. Quantitative analysis of the scratch area indicated that OA inhibits the migration of Huh-7 cells (*p* < 0.05) (Figure 9a,b).

#### 2.2.6. Effect of OA on the mRNA Expression of Lipid Metabolism-Related Genes in Huh-7 Cells

**Figure 10:**
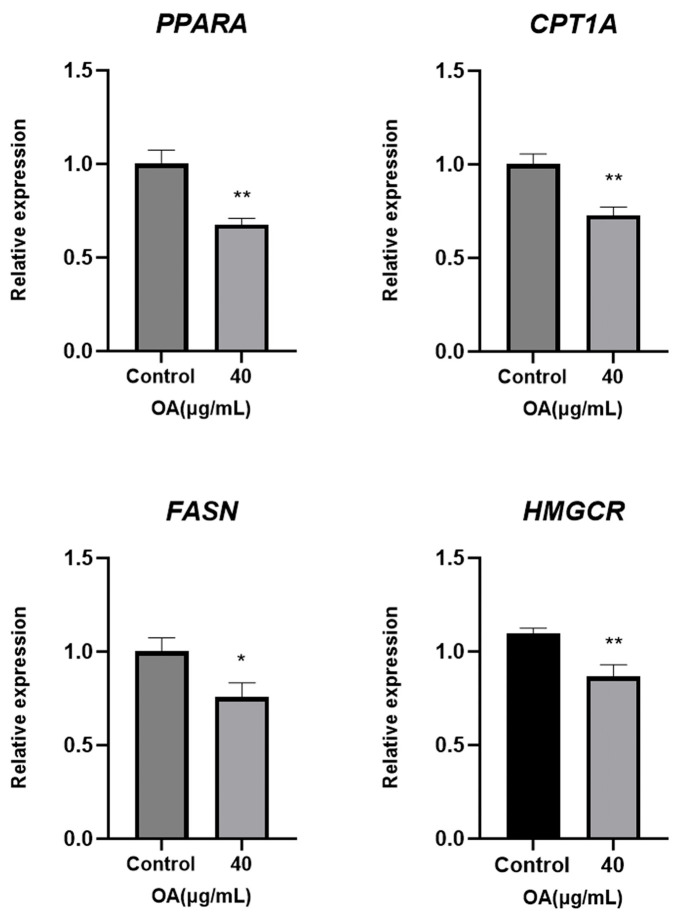
The relative expression of mRNA of *PPARA*, *CPT1A*, *FASN* and *HMGCR* in Huh-7 cells from the Control and OA 40 μg/mL. Cells were treated with OA at the indicated concentrations for 24 h. Data are presented as the mean ± SD (*n* = 3 independent replicates per group). * *p* < 0.05, ** *p* < 0.01 vs. the Control group. The experiment was independently repeated three times.

The experimental data indicated that the treatment group (40 μg/mL OA) exhibited a significant reduction in mRNA expression levels of *PPARA* (*p* < 0.01), Carnitine Palmitoyltransferase 1A (*CPT1A)* (*p* < 0.01), Fatty Acid Synthase (*FASN)* (*p* < 0.05) and *HMGCR* (*p* < 0.01) compared to the Control group (Figure 10).

#### 2.2.7. Effect of OA on Protein Expression Levels in Huh-7 Cells

**Figure 11:**
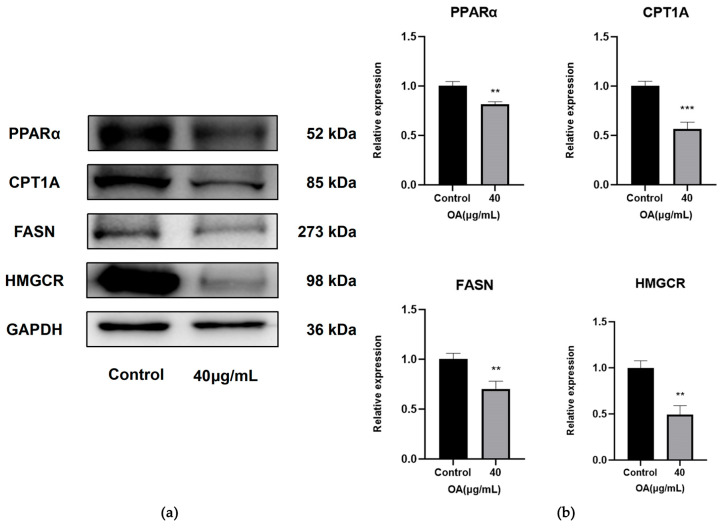
Expression levels of proteins related to lipid metabolism. (**a**) Protein expression levels of PPARα, CPT1A, FASN and HMGCR in Huh-7 cells from the Control and OA 40 μg/mL. (**b**) Statistical analysis of relative protein expression levels determined by Western blotting. GAPDH was used as an internal control. Cells were treated with OA at the indicated concentrations for 24 h. Data are presented as the mean ± SD (*n* = 3 independent replicates per group). ** *p* < 0.01, *** *p* < 0.001 vs. the Control group. The experiment was independently repeated three times.

The experimental results presented in the figure demonstrate that, in comparison to the Control group, the treatment group (40 μg/mL OA) shows a significant reduction in the protein expression levels of PPARα, CPT1A, FASN and HMGCR (*p* < 0.01) (Figure 11a,b).

#### 2.2.8. Summary

In summary, OA attenuated intracellular lipid accumulation, suppressed cellular migration, and downregulated the expression of genes associated with lipid metabolism. By concurrently inhibiting both fatty acid oxidation (FAO) and fatty acid synthesis (FAS), OA disrupted lipid metabolism in HCC cells, ultimately restraining both cellular proliferation and migration.

### 2.3. Effect of OA on HCC via the PPARα-CPT1A Axis

Given that OA modulated lipid metabolism, we focused on the PPARα–CPT1A axis as a crucial lipid metabolic pathway. To ascertain whether OA acts through this pathway, we utilized the PPARα-specific antagonist GW6471 and the agonist Pemafibrate. GW6471 binds to PPARα, stabilizing it in a transcriptionally repressive state [12], while Pemafibrate binds via a distinct Y-shaped ligand-binding domain [13].

#### 2.3.1. In Vivo Experiments

##### Inhibitory Effect of OA on the Growth of H22 Xenograft Tumors

**Figure 12:**
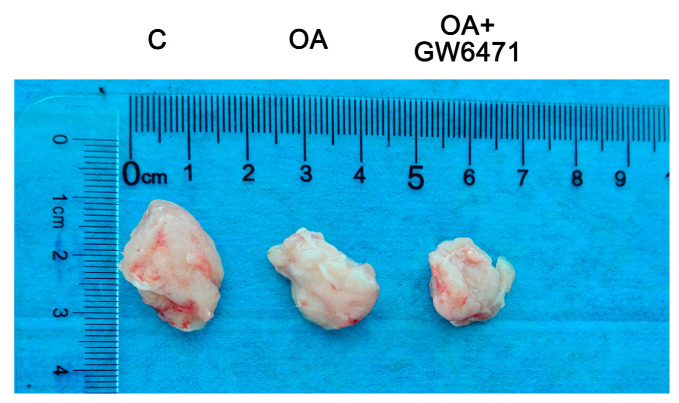
Tumor tissues from different groups: C: model control group; OA: Oleanolic acid monotherapy group; OA + GW6471: Oleanolic acid + GW6471 combination therapy group.

Oral administration of OA (100 mg/kg/day) [14] significantly inhibited the growth of H22 hepatoma allografts, achieving a tumor growth inhibition rate of 31.16% compared to the Control group. When combined with the PPARα inhibitor GW6471, the inhibition rate increased to 40.04%, a value that is significantly higher than that observed with OA alone. These findings suggest that OA effectively suppresses tumor growth in vivo, and the co-administration of GW6471 further enhanced its therapeutic efficacy (Figure 12 and Table 3).

##### Effects of OA on Tumor Histomorphology in BALB/c Mice Bearing H22 Xenografts

**Figure 13:**
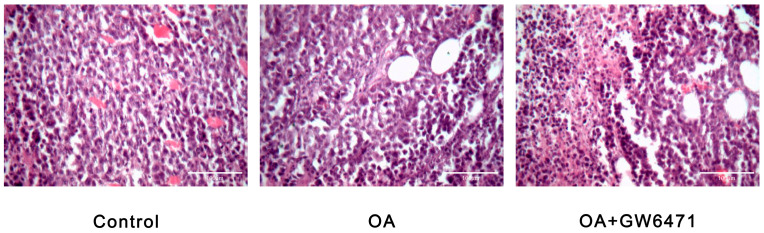
Effects of OA on tumor histomorphology in BALB/c mice bearing H22 xenografts (bar = 100 μm).

OA treatment significantly decreased both tumor volume and weight in mice bearing H22 xenografts. Histological analysis revealed that tumors in the model group exhibited densely arranged round-to-oval cells, characterized by vigorous growth, frequent mitotic activity, abundant vasculature, and invasive growth patterns extending to the sternum and clavicle. In contrast, tumors treated with OA displayed sparse cellular arrangement, pronounced degeneration and necrosis, lymphocyte infiltration, reduced vascular density, and vacuolation, with the most significant effects observed in the combination treatment group (Figure 13).

##### Effect of OA on Protein Expression Levels in H22 Xenograft Tumors

**Figure 14:**
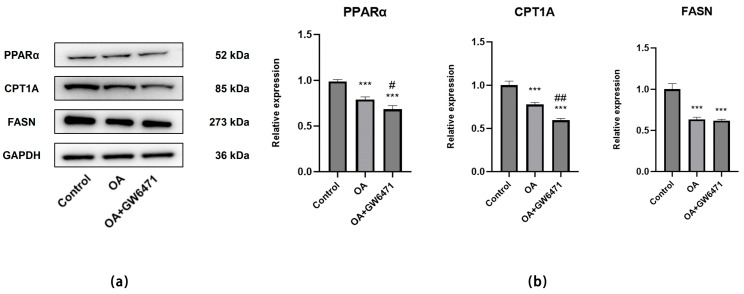
Expression levels of proteins related to lipid metabolism. (**a**) Protein expression levels of PPARα, CPT1A and FASN in H22 xenograft tumor tissues with Control, OA (100 mg/kg/day) and OA + GW6471 (100 mg/kg/day OA + 5 mg/kg/day GW6471). (**b**) Statistical analysis of relative protein expression levels determined by Western blotting. GAPDH was used as an internal control. Data are presented as the mean ± SD (*n* = 3 independent replicates per group). *** *p* < 0.001 vs. the Control group. ^#^ *p* < 0.05, ^##^ *p* < 0.01 vs. the OA group. The experiment was independently repeated three times.

Compared to the Control group, both the OA-treated group and the OA plus GW6471 combination group exhibited significant reductions in the protein expression levels of PPARα, CPT1A and FASN (*p* < 0.05). In comparison to OA alone, the combination treatment resulted in a further decrease in PPARα and CPT1A expression (*p* < 0.05); however, no statistically significant difference was observed in FASN expression (*p* > 0.05) (Figure 14a,b).

##### Summary

These results indicate that OA inhibited the growth of HCC in vivo by influencing the PPARα–CPT1A axis, and that the inhibition of PPARα enhances this effect.

#### 2.3.2. In Vitro Experiments

##### Effects of Inhibiting the PPARα-CPT1A Axis on Huh-7 Cells

**Figure 15:**
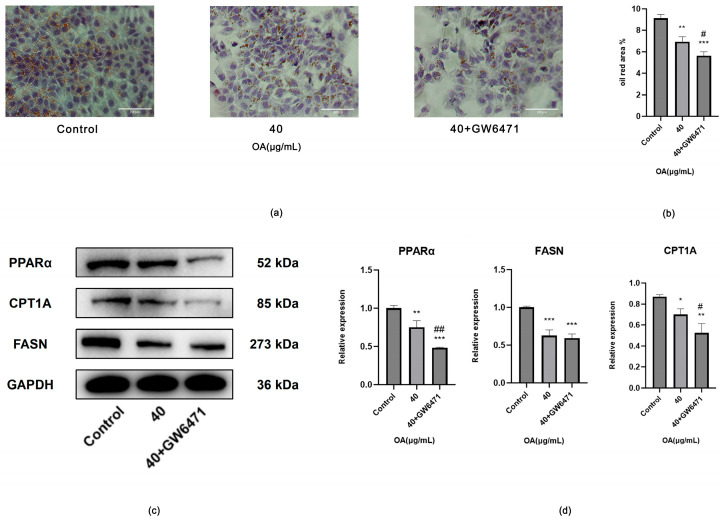
(**a**) Representative images of Oil Red O staining in Huh-7 cells treated with Control, 40 μg/mL OA, and the combination of OA and GW6471 (40 μg/mL OA + 1 μM GW6471) (bar = 100 μm). (**b**) Statistical analysis results of Oil Red O-stained images. Cells were treated at the indicated concentrations for 24 h. Data are presented as the mean ± SD (*n* = 3 independent replicates per group). ** *p* < 0.01, *** *p* < 0.001 vs. the Control group. ^#^ *p* < 0.05 vs. the OA group. The experiment was independently repeated three times. (**c**) Protein expression levels of PPARα, CPT1A and FASN in Huh-7 cells from the Control, 40 μg/mL OA, and the combination of OA and GW6471 (40 μg/mL OA + 1 μM GW6471). (**d**) Statistical analysis of relative protein expression levels determined by Western blotting. GAPDH was used as an internal control. Cells were treated at the indicated concentrations for 24 h. Data are presented as the mean ± SD (*n* = 3 independent replicates per group). * *p* < 0.05, ** *p* < 0.01, *** *p* < 0.001 vs. the Control group. ^#^ *p* < 0.05, ^##^ *p* < 0.01 vs. the OA group. The experiment was independently repeated three times.

OA markedly reduced the number and diameter of lipid droplets in Huh-7 cells. The combination therapy demonstrated a greater improvement compared to OA alone, indicating a more pronounced attenuation of lipid deposition (*p* < 0.05) (Figure 15a,b).

In comparison to the Control, both OA alone and the combination of OA with GW6471 significantly decreased the protein levels of PPARα, CPT1A and FASN (*p* < 0.05). Relative to OA alone, the combination therapy further reduced the levels of PPARα and CPT1A (*p* < 0.01), while FASN did not show a significant difference (*p* > 0.05) (Figure 15c,d).

##### Effects of Activating the PPARα-CPT1A Axis on Huh-7 Cells

**Figure 16:**
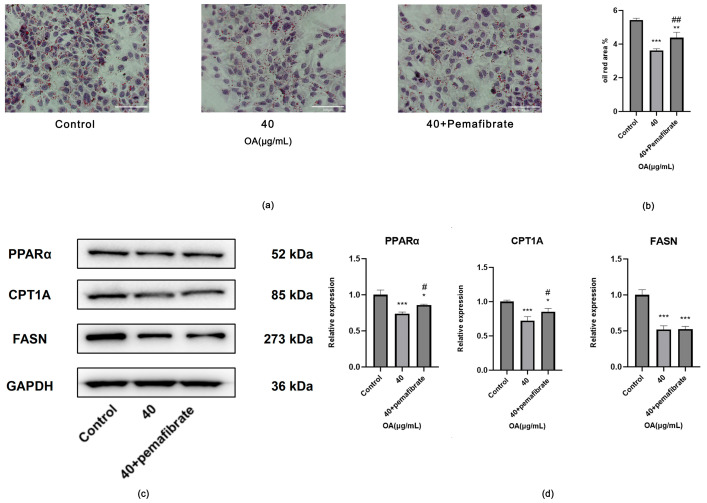
(**a**) Representative images of Oil Red O staining in Huh-7 cells treated with Control, 40 μg/mL OA, and the combination of OA and Pemafibrate (40 μg/mL OA + 50 nM Pemafibrate) (bar = 100 μm). (**b**) Statistical analysis results of Oil Red O-stained images. Cells were treated at the indicated concentrations for 24 h. Data are presented as the mean ± SD (*n* = 3 independent replicates per group). ** *p* < 0.01, *** *p* < 0.001 vs. the Control group. ^##^ *p* < 0.01 vs. the OA group. The experiment was independently repeated three times. (**c**) Protein expression levels of PPARα,CPT1A and FASN in Huh-7 cells from the Control, 40 μg/mL OA, and the combination of OA and Pemafibrate (40 μg/mL OA + 50 nM Pemafibrate) (**d**) Statistical analysis of relative protein expression levels determined by Western blotting. GAPDH was used as an internal control. Cells were treated at the indicated concentrations for 24 h. Data are presented as the mean ± SD (*n* = 3 independent replicates per group). * *p* < 0.05, *** *p* < 0.001 vs. the Control group. ^#^ *p* < 0.05 vs. the OA group. The experiment was independently repeated three times.

Compared to the Control group, OA monotherapy significantly reduced both the number of lipid droplets and their diameter. In contrast, combination therapy led to an increase in lipid droplets when compared to OA monotherapy (*p* < 0.05) (Figure 16a,b).

Compared to the Control group, OA treatment significantly reduced the protein expression levels of PPARα, CPT1A, and FASN (*p* < 0.05). In contrast to OA alone, the combination treatment resulted in an upregulation of the protein expression levels of PPARα and CPT1A (*p* < 0.05), while no statistically significant difference was found in the protein expression levels of FASN (*p* > 0.05) (Figure 16c,d).

##### Summary

OA monotherapy resulted in a reduction in lipid droplet accumulation and suppressed the expression of PPARα, CPT1A and FASN. Inhibition of PPARα enhanced these effects, while agonism of PPARα negated them. These findings indicate that OA modulated lipid metabolism in HCC through the PPARα–CPT1A axis.

## 3. Discussion

Most antitumor drugs are prone to inducing liver injury, which constitutes a significant global public health issue and leads to drug withdrawal and late-stage R&D failure, posing considerable challenges to the medical field. In this context, antitumor agents that combine therapeutic efficacy with pronounced hepatoprotective properties offer broader development prospects by simultaneously addressing the dual core demands of treatment efficacy and safety, emerging as a pivotal research direction. Notably, the liver-protective drug OA has demonstrated substantial therapeutic value in managing liver diseases, with proven efficacy against various prevalent hepatic disorders, including MASLD, HCC and liver fibrosis, thereby providing an effective therapeutic option for liver conditions [15]. This study is the first to focus on the relationship between OA and lipid metabolism in liver cancer. Through network pharmacology screening (Figure 1 and Figure 2), molecular docking (Figure 3 and Table 1), and MD simulation prediction (Figure 4 and Figure 5 and Table 2), we concluded that OA alleviates lipid accumulation and inhibits proliferation by suppressing the expression of lipid metabolism-related proteins such as PPARα, while reducing FAS and FAO in liver cancer cells.

PPARα is highly expressed in the liver and serves as a key regulator of hepatic lipid metabolism, which may be closely associated with liver cancer [16,17]. It regulates lipid and energy homeostasis in the liver through three FAO metabolic pathways: mitochondrial β-oxidation, peroxisomal β-oxidation and microsomal ω-oxidation [18]. PPARα exhibits a dual role in tumor therapy; studies have shown that its activation can induce endogenous apoptosis in tumor cells and inhibit glycolysis, thereby exerting anti-tumor effects [19]. Additionally, PPARα activation can inhibit tumor angiogenesis and suppress the growth and metastasis of primary cancers [20]. In the present study, OA exerted anti-tumor effects by inhibiting PPARα activity (Figure 10 and Figure 11), suppressing cell migration (Figure 9), and promoting tumor cell apoptosis (Figure 7). Notably, other studies have reported the cancer-promoting role of PPARα in tumors: the use of PPARα-specific antagonists (such as GW6471 and TPST-1120) can induce apoptosis, reverse the immunosuppressive microenvironment, and ultimately inhibit tumor cell growth [21]. Furthermore, activation of the PPARα pathway can lead to tumor cell drug resistance, while inhibition of PPARα activity can reverse this resistance and restore cell sensitivity to cetuximab [22].

CPT1A serves as the key rate-limiting enzyme in mitochondrial fatty acid β-oxidation and facilitates the transport of long-chain fatty acids into the mitochondrial matrix for oxidative degradation [23]. PPARα directly regulates CPT1A expression, establishing a positive regulatory relationship [24]. Consequently, PPARα and CPT1A together form the core regulatory axis responsible for maintaining lipid metabolism homeostasis. FASN, the rate-limiting enzyme in de novo FAS, governs endogenous fatty acid production [25]. In the present study, OA treatment significantly downregulated the mRNA and protein levels of *PPARA*, *CPT1A* and *FASN*. Concurrently, Oil Red O staining (Figure 8a,b), along with TG quantification (Figure 8c), confirmed a reduction in intracellular lipid content. Superficially, the reduced expression of PPARα and CPT1A would be expected to inhibit FAO, which might lead to lipid accumulation. However, the present study did not observe such a phenomenon; instead, a decrease in total lipid content was noted. We propose that this discrepancy arises because OA simultaneously disrupts both the synthetic source and the degradative pathway of fatty acids. Under these conditions, cells are unable to compensate for lipid loss through endogenous synthesis or exogenous uptake.

To further validate the role of OA in regulating lipid metabolism in liver cancer via PPARα, we employed the PPARα-specific agonist pemafibrate and the inhibitor GW6471 for intervention. The results from Western blotting analysis demonstrated that the combination of pemafibrate and OA effectively reversed the OA-induced downregulation of PPARα and CPT1A protein expression. Conversely, treatment with GW6471 further suppressed the expression levels of both PPARα and CPT1A (Figure 14, Figure 15 and Figure 16). In conclusion, PPARα emerges as a critical regulatory node through which OA modulates lipid metabolism in liver cancer.

Based on these findings, we propose that the broader systemic metabolic effects of OA via the PPARα-CPT1A axis may underlie a potential regulatory mechanism linking the liver and spleen. Studies have demonstrated that OA not only regulates hepatocyte lipid metabolism but also serves as a pivotal hub for metabolic reprogramming. By activating the PPARα-CPT1A axis, OA may systematically reshape the splenic metabolic microenvironment, potentially transforming it from a passive accumulation site for metabolic waste into an efficient energy utilization engine. This transformation yields a triple synergistic benefit: it clears toxic lipids, suppresses chronic inflammation, and prevents tissue fibrosis [26]. This molecular recalibration preserves the dual function of the spleen as both an immune surveillance barrier and an organ of metabolic homeostasis. More importantly, this mechanism enhances the functional connection within the ‘liver–spleen axis’ [27]. The lipid clearance driven by OA in the liver reduces the levels of pro-inflammatory cytokines and metabolic toxins transported to the spleen via the portal vein, thereby coordinating systemic metabolic filtration and immune defense, and enhancing the body’s overall resistance to metabolic stress.

**Figure 17:**
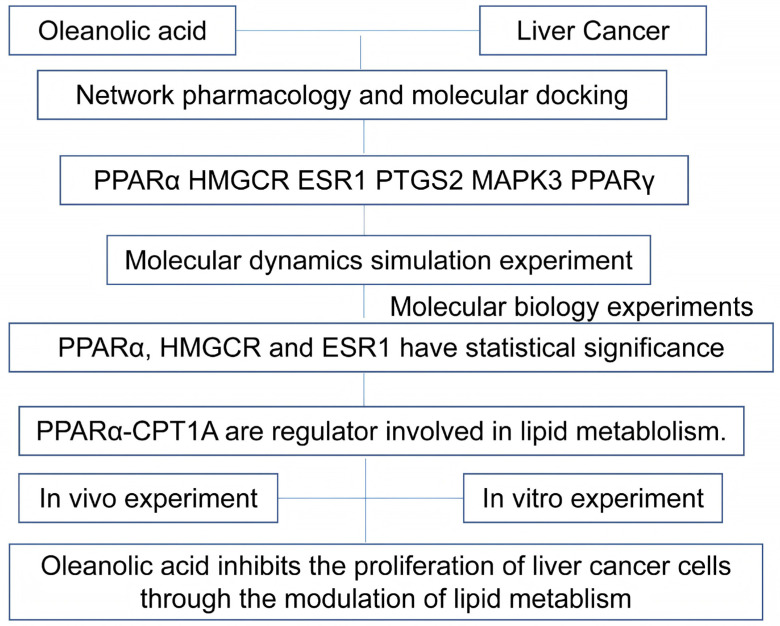
Workflow of the study. The flowchart summarizes the overall experimental design and analytical approach, including network pharmacology analysis, molecular docking, molecular dynamics simulations, in vitro cellular assays, and in vivo animal experiments using an H22 xenograft mouse model.

This study confirmed through bioinformatics predictions and both in vivo and in vitro experiments that OA regulates lipid metabolism and inhibits the proliferation of HCC cells by affecting the PPARα-CPT1A axis. However, certain limitations must be acknowledged: (i) The in vitro experiments were conducted using conventional 2D monolayer cell culture systems, which lack key characteristics of a 3D microenvironment, such as cell–extracellular matrix interactions, nutrient/oxygen gradients, and drug penetration barriers. Therefore, the regulatory effects of OA on lipid metabolism in more physiologically relevant 3D culture models (e.g., organoids or co-culture systems) that better mimic in vivo conditions require further validation in subsequent studies. (ii) Given the clinical potential of the PPARα inhibitor TPST-1120 in HCC treatment, this study explored the inhibitory effects of the traditional Chinese medicine natural extract OA on Huh-7 and H22 HCC cells. However, due to the relatively limited range of cell lines employed, this research has inherent limitations. Future studies should incorporate a broader variety of HCC cell lines to systematically and comprehensively elucidate the mechanism of OA’s effects on liver cancer, thereby laying a robust experimental foundation for its clinical application. (iii) The mechanisms influencing the progression of liver cancer are complex, involving dynamic shifts in energy sources. Lipid metabolism represents only one aspect, alongside pathways such as glucose metabolism and amino acid metabolism. This study focused solely on lipid metabolism to investigate OA’s inhibitory effects on liver cancer. Future research should consider the roles of other metabolic pathways in tumor progression, as well as their mutual regulation and interactions, to further refine the understanding of OA’s anti-HCC mechanisms (Figure 17).

## 4. Materials and Methods

### 4.1. Materials

#### 4.1.1. Experimental Reagents

The following materials were utilized in this study: OA (Shanghai Yuanye Biotechnology, Shanghai, China, B20954), Huh-7-specific culture medium (Wuhan Puno Biotechnology, Wuhan, China, CM-0120), PBS, 0.25% trypsin digestion solution, and CCK-8 assay kit (Dalian Meilun Biotechnology, Dalian, China, MA0010, MA0234, MA0218), RNAeasy™ Animal RNA Extraction Kit (Centrifugal Column) (Biotree, Shanghai, China, R0027), BeyoRT™ III cDNA First Strand Synthesis Premix (5X) (Biotree, Shanghai, China, D7182M), ChamQ Universal SYBR qPCR MasterMix (Nanjing Novoprotein, Nanjing, China, Q711-02), TG Reagent Kit (Nanjing Jiancheng, Nanjing, China, A110-1-1), PPARα and FASN antibodies (Genuin Biotech, Hefei, China, 52,637 and 4278), CPT1A and HMGCR antibodies (Youpin Biologicals, Hangzhou, China, YP-mAb-08231 and YP-mAb-17137), GAPDH antibody (Biyuntian, Shanghai, China, AF1186), horseradish peroxidase (HRP)-conjugated goat anti-mouse IgG (H+L) and rabbit anti-goat IgG (H+L) (Biyuntian, Shanghai, China, A0216 and A0208), Hoechst 33342/PI Staining Kit (Yeasen Biotech, Shanghai, China, 40744ES), GW6471 and Pemafibrate (AbMole, Shanghai, China, M2746 and MM10245).

#### 4.1.2. Cell Lines

The Huh-7 human liver cancer cell line and the H22 liver cancer cell line were acquired from Haixing Biotechnology (Suzhou, China), whereas the THLE-2 liver cell line was purchased from Procell (Wuhan, China).

#### 4.1.3. Databases

All databases utilized in this study are outlined in Table 4.

### 4.2. Experimental Methods

#### 4.2.1. Computational Simulation Methods

##### Network Pharmacology

Gathering and Screening for OA Targets

The molecular structure of OA was retrieved from the PubChem database (Table 4). In the SwissTargetPrediction database (Table 4), we established a threshold of Probability ≥ 0.1 [28]. This threshold represents the default value recommended by the platform, which is intended to balance prediction sensitivity with the false positive rate.

Collection of Liver Cancer-Related Targets

Using the OMIM and GeneCards databases (Table 4), searches were conducted with the keyword “liver cancer” [29,30,31]. The median method was employed to extract liver cancer targets from both databases. Subsequently, these targets were consolidated, and redundant values were eliminated to create a comprehensive set of liver cancer targets. A Venn analysis of OA targets and liver cancer targets was performed using the Venny 2.1.0 online tool (Table 4) to identify intersecting targets, thereby revealing the potential therapeutic targets of OA in HCC.

Development of the PPI Network

The identified overlapping genes were uploaded to the STRING database (Table 4) for interaction visualization. In this study, the species was classified as Homo sapiens, and the threshold for interactions was set to a medium confidence value, specifically greater than 0.4 [32]. This threshold is the default setting recommended by the STRING platform (Table 4) and has been widely adopted in numerous network pharmacology studies, underscoring its relevance and reliability in the field. The resultant TSV file was then imported into Cytoscape 3.9.1 for network visualization [33].

GO and KEGG Enrichment Analysis

To further investigate the biological processes and signaling pathways involved in the therapeutic effects of OA on liver cancer, we analyzed the identified intersecting genes using the DAVID database [34]. The results were visualized using a microbioinformatics website (Table 4).

##### Molecular Docking

The identified key potential targets underwent molecular docking studies in conjunction with OA. The structure of OA was obtained from PubChem (Table 4), while the protein structures were retrieved from the RCSB Protein Data Bank (PDB). Water molecules and original ligands were removed using PyMOL (2.6). Hydrogen atoms, charges, and atom types were added using AutoDockTools (1.5.7), and the structures were subsequently saved in PDBQT format. The docking box was centered on the geometric center of each protein ligand-binding domain, with dimensions designed to ensure complete coverage of the active pocket. Given the polycyclic rigid structure of OA, a global search strategy was employed. The exhaustiveness parameter was set to the default value of 8 [35], and docking scores were reported as scoring function values in -kcal/mol. Finally, molecular docking calculations were performed using AutoDock Vina (1.2) [36], and the docking results were visualized with PyMOL software to analyze the binding affinity between the drug and its protein target.

##### MD Simulations

Based on the docking results, classical MD simulations were conducted using GROMACS (version 2023.5) to validate the binding of each ligand to the target protein [37]. The simulations utilized binding site coordinates obtained from docking, with a duration of 100 ns. The protein PDB files were processed to generate the corresponding topology, and the ligand topology was created using ACPYPE, which was then integrated into the protein topology to construct the complex file [38]. The AMBER14SB force field was employed to describe the proteins [39], demonstrating significant improvements in maintaining the accuracy of backbone and side-chain conformations compared to previous versions. The TIP3P explicit water model was utilized for water molecules [40], as it exhibits good compatibility with the AMBER force field and high computational efficiency. The system was placed in a cubic water box with an edge length of 1.0 nm, followed by the addition of water molecules and Na^+^/Cl^−^ ions to neutralize the charge and prevent steric hindrance between the protein and ligand. Energy minimization was performed using the steepest descent method at 300 K. Subsequently, the temperature was gradually increased from 0 K to 300 K under positional restraints. Short-range electrostatic interactions and van der Waals forces were treated using the Particle Mesh Ewald (PME) method. The system temperature was maintained at 300 K using the velocity-rescale thermostat, while the pressure was regulated at 1 bar using the Parrinello–Rahman barostat. Position-restrained MD simulations were conducted for 50,000 steps (with a time step of 2 fs) under both NVT and NPT conditions. These two equilibration phases are standard practices in MD simulations aimed at stabilizing the system’s temperature and pressure. Subsequently, a 100 ns molecular dynamics simulation was conducted on the complex system [41], utilizing a time step of 2 fs and capturing one frame every 10 ps. The generated trajectories were analyzed for RMSD, RMSF and other relevant metrics. To evaluate binding energetics and intermolecular interactions, the gmx_MMPBSA.py script was employed to perform MM-PBSA analysis, thereby inferring the binding mode between the ligands and proteins. The energy terms reported in the MM-PBSA calculations are as follows:

ΔG_complex: total free energy of the protein–ligand complex;

ΔG_protein and ΔG_ligand: free energies of the protein and ligand in solvent;

ΔVDWAALS: van der Waals interactions;

ΔEEL: electrostatic interactions;

ΔEPB: polar solvation energy (PB method);

ΔENPOLAR: polar contributions to solvation energy (solute–solvent interactions in PB);

ΔEDISPER: dispersion energy (nonpolar solute–solvent attractions);

ΔGGAS: gas-phase MM energy (bonded and nonbonded terms);

ΔGSOLV: total solvation energy (PB-based);

ΔTOTAL: total binding-related energy, all values are reported in kcal/mol.

#### 4.2.2. Cellular Experiment

##### Preparation of OA Solution

OA (purity ≥ 98%) was dissolved in dimethyl sulfoxide (DMSO) and stored at −20 °C. For experimental procedures, the stock solution was diluted with culture medium to achieve the desired concentrations.

##### Cell Culture

Huh-7 liver cancer cells were routinely cultured in Huh-7-specific medium at 37 °C with 5% CO_2_. THLE-2 cells were cultured in DMEM supplemented with 20% FBS under identical conditions. When the cells reached approximately 80% confluence, those in the exponential growth phase were harvested for subsequent experiments.

##### CCK-8 Assay for Cell Proliferation

Cells in the exponential growth phase were resuspended and seeded into 96-well plates at a density of 4 × 10^3^ cells per well. The plates were incubated for 24 h. The following day, the culture medium was discarded, and the cells were treated with OA at concentrations of 30, 35, 40, 45, 50, 55, 60 and 65 μg/mL for 24 h. Subsequently, 10 μL of CCK-8 reagent was added to each well, and the plates were incubated in the dark for an additional 2 h. The optical density (OD) was measured at 450 nm using a microplate reader to assess cell viability. The cell survival rate was calculated as follows:

Cellviability(%)=(TreatmentOD−BlankOD)/(ControlOD−BlankOD)×100%

##### Screening of OA Concentration by Hoechst 33342/PI Double Staining

Cells in the exponential growth phase were harvested and seeded into 96-well plates at a density of 4 × 10^3^ cells per well. The plates were incubated for 24 h, after which the culture medium was aspirated and replaced with fresh medium containing OA at concentrations of 20, 40 and 60 μg/mL. A vehicle control group was included for comparison. All groups were then incubated for an additional 24 h. Following this incubation, the medium was discarded, and the cells were gently rinsed once with PBS. A staining working solution was prepared by mixing Hoechst 33342 and PI with staining buffer at a ratio of 5 μL:5 μL:1 mL. Subsequently, 100 μL of this working solution was added to each well, and the plates were incubated for 15 min. Finally, the plates were transferred to a fluorescence microscope for observation and image acquisition, all completed within 1 h.

##### Oil Red O Staining for Lipid Droplet Detection in Cells

Cells in the exponential growth phase were seeded at a density of 2 × 10^5^ cells per well in a 12-well plate. After 24 h, the cells were divided into the Control group (0 μg/mL OA) and the treatment group (40 μg/mL OA). Both groups were then incubated for an additional 24 h. The culture medium was subsequently discarded, and the cells were washed with PBS. Cells were fixed with 4% paraformaldehyde for 25 min. Following fixation, the cells were briefly rinsed with 60% isopropanol for 15 s and then stained with Oil Red O solution for 25 min. After staining, the cells were rinsed with 60% isopropanol to remove excess dye and then counterstained with hematoxylin for 1 min. After thorough washing, the samples were stored in PBS for microscopic observation.

##### GPO-PAP Method for Intracellular TG Content Determination

Cells in the exponential growth phase were seeded into T25 culture flasks at a density of 1.2 × 10^6^ cells per flask. After 24 h of incubation, cells from both the Control group (0 μg/mL OA) and the treatment group (40 μg/mL OA) were collected. The samples were centrifuged at 1000 r/min for 10 min. Following the removal of the supernatant, each pellet was washed with PBS and centrifuged again; this washing step was repeated once, resulting in a total of two washes. The cell pellets were then resuspended in 0.3 mL of PBS. Subsequently, the samples were placed on ice and disrupted by ultrasonication to obtain homogeneous lysates for further analysis.

##### Scratch Assay to Detect the Migration Ability of Huh-7 Cells

Cells in the exponential growth phase were harvested and uniformly seeded into 6-well plates at a density of 5 × 10^5^ cells per well. The plates were incubated for 24 h at 37 °C in a humidified atmosphere containing 5% CO_2_. Once the cells reached approximately 90% confluence, the culture medium was aspirated, and the adherent cells were gently rinsed twice with PBS. Each well was then replenished with 2 mL of serum-free DMEM. This serum starvation step was performed for 12 h to induce cell cycle synchronization at the G0/G1 phase, thereby inhibiting cellular proliferation. A sterile 200 μL pipette tip was used to create a straight, linear scratch perpendicular to the bottom of the plate. Following the generation of the scratch, each well was carefully rinsed with PBS to remove detached cells and debris, and fresh culture medium was added. Bright-field images of the scratches were immediately captured at the 0 h time point using an inverted light microscope. The cells were then assigned to two experimental groups: the Control group (0 μg/mL OA) and the treatment group (40 μg/mL OA). All plates were returned to the incubator for an additional 24 h. After this incubation period, images were again acquired. The scratch wound areas at 0 h and 24 h were quantified using ImageJ(1.54p) software to calculate the cell migration rate. The percentage of cell migration was calculated using the following formula: Migration rate (%) = [(A0 − A24)/A0] × 100%, where A0 represents the initial wound area at 0 h and A24 represents the remaining wound area after 24 h.

##### qRT-PCR for Gene Expression Analysis

Huh-7 cells in the exponential growth phase were seeded into 6-well plates. After 24 h, the cells were treated with the Control group (0 μg/mL OA) and the treatment group (40 μg/mL OA) for an additional 24 h. Following treatment, the culture medium was discarded, and the cells were collected. Total RNA was extracted using the RNAeasy™ Animal RNA Extraction Kit. Reverse transcription was conducted using the BeyoRT™ III cDNA First Strand Synthesis Premix (5X) to synthesize cDNA. qPCR was performed using ChamQ Universal SYBR qPCR MasterMix on a real-time PCR system model. The thermal cycling conditions were as follows: initial denaturation at 95 °C for 10 min, followed by 40 cycles of 95 °C for 30 s, 56 °C for 30 s, and 72 °C for 45 s. A melt curve analysis was conducted after amplification under the following conditions: 95 °C for 15 s, 60 °C for 1 min, and 95 °C for 15 s. GAPDH served as an internal control. Relative gene expression levels were calculated using the 2^−ΔΔCt^ method. The primer sequences are listed in Table 5.

##### Western Blotting Analysis of Protein Expression

Huh-7 cells were divided into the Control group (0 μg/mL OA) and the treatment group (40 μg/mL OA) for 24 h. Cellular proteins were extracted using SDS lysis buffer. Protein separation was performed via electrophoresis utilizing an SDS-PAGE rapid gel preparation kit, followed by the transfer of separated proteins onto PVDF membranes. The membranes were subsequently blocked with 5% nonfat dry milk. After blocking, the membranes were washed and incubated with primary antibodies against FASN (1:5000), PPARα (1:2000), CPT1A (1:2000), HMGCR (1:1000), and GAPDH (1:5000) as a loading control. Following this, the membranes were incubated with HRP-conjugated goat anti-mouse IgG (H + L) or HRP-conjugated goat anti-rabbit IgG (H + L) at a dilution of 1:1000, depending on the species of the primary antibody. Protein bands were visualized using a Tanon chemiluminescence imaging system.

##### Oil Red O Staining for Detection of Lipid Droplets in Cells

In the antagonist inhibition experiment, Huh-7 cells were divided into three groups: the Control group (0 μg/mL OA), the treatment group (40 μg/mL OA), and the combination group (40 μg/mL OA + 1 µM GW6471). Similarly, in the agonist rescue experiment, Huh-7 cells were also divided into three groups: the Control group (0 μg/mL OA), the treatment group (40 μg/mL OA), and the combination group (40 μg/mL OA + 50 nM Pemafibrate). After a 24 h treatment period, the cells were processed following the methodology outlined above for Oil Red O Staining for Lipid Droplet Detection in Cells.

##### Western Blotting Analysis of Protein Expression

In the antagonist inhibition experiment, Huh-7 cells were categorized into three groups: the Control group (0 μg/mL OA), the treatment group (40 μg/mL OA), and the combination group (40 μg/mL OA + 1 µM GW6471). In the agonist rescue experiment, Huh-7 cells were also divided into three groups: the Control group (0 μg/mL OA), the treatment group (40 μg/mL OA), and the combination group (40 μg/mL OA + 50 nM Pemafibrate). After a 24 h treatment period, the cells were processed following the methodology outlined above for Western Blotting Analysis of Protein Expression.

#### 4.2.3. Vivo Experiments

##### Experimental Animals

A total of 28 eight-week-old, specific-pathogen-free (SPF) BALB/c mice (18–22 g; 14 males and 14 females) were procured from Liaoning Changsheng (SCXK2025-0001). The mice were maintained in the animal facility of the Scientific Experiment Center at Heilongjiang University of Chinese Medicine, with the room temperature regulated between 20 and 24 °C. This experiment adhered strictly to the standards outlined in the “Guide for the Care and Use of Laboratory Animals.” The study protocol received approval from the Chinese Medicine Institutional Animal Care and Use Committee of Heilongjiang University (No. 2025111701).

##### Modeling and Grouping

H22 hepatocarcinoma cells were maintained through intraperitoneal passage in BALB/c mice. Upon abdominal distension, the mice were euthanized, and milky ascitic fluid was aseptically collected. The ascitic fluid was washed twice with saline, centrifuged, and resuspended to obtain a single-cell suspension. Trypan blue staining confirmed a cell viability exceeding 95%. The cell concentration was adjusted to 1 × 10^7^ cells/mL using saline. Subsequently, 0.2 mL of this suspension was inoculated subcutaneously into the right axilla of each mouse to establish the H22 transplanted tumor model. Once the model was successfully established, the mice were randomly divided into three groups (*n* = 6 per group): a Control group, an OA group (100 mg/kg/day) and a combination group (OA 100 mg/kg/day plus GW6471 5 mg/kg/day).

##### Drug Administration

Following the successful establishment of the model, the model control group was administered daily intragastric doses of normal saline, matching the volume given to the treatment groups. The OA group received OA at a dosage of 100 mg/kg/day via gavage once daily. The combination group was treated with OA at the same dosage of 100 mg/kg/day by gavage daily, in conjunction with an intraperitoneal injection of GW6471 at a dosage of 5 mg/kg/day. All groups underwent continuous treatment for a duration of 14 days.

##### Light Microscopy for Morphological Observation of Tumor Cells

Tumor tissues were fixed in 4% paraformaldehyde. After dehydration, clearing, and paraffin embedding, the sections were stained with hematoxylin and eosin (H&E), mounted, and examined under a light microscope.

##### Western Blotting Analysis of Protein Expression

Tumor tissue samples were rapidly excised on ice. A 100 mg portion of each sample was weighed and placed into a 1.5 mL microcentrifuge tube. SDS lysis buffer was added at a ratio of 100 μL per 1 mg of tumor tissue. The tissues were thoroughly homogenized on ice using an ultrasonic homogenizer until no visible fragments remained. The homogenate was then centrifuged at 12,000 rpm for 10 min. The supernatants were collected and transferred into a new microcentrifuge tube for storage at −80 °C. Protein concentrations were determined using a BCA assay, with absorbance measured at 595 nm using a microplate reader. All samples were adjusted to the same protein concentration using lysis buffer based on the calculated protein content. Subsequent procedures were performed as described in Section Western Blotting Analysis of Protein Expression.

### 4.3. Statistical Analysis

All statistical analyses were conducted using GraphPad Prism 10. Two-group comparisons were assessed using two-tailed unpaired *t*-tests. For comparisons involving three or more groups, one-way ANOVA was utilized, followed by Tukey’s multiple comparison test for post hoc pairwise comparisons. When the data did not conform to a normal distribution, as assessed by the Shapiro–Wilk test, nonparametric tests were employed. A significance level of *p* < 0.05 was established, *p* > 0.05 indicating no significant difference.

## 5. Conclusions

In summary, this study confirmed that OA modulated lipid metabolism in HCC by targeting PPARα as a central regulatory factor, both in vitro and in vivo. Consequently, OA presents a promising novel therapeutic strategy for the treatment of liver cancer.

## Figures and Tables

**Table 1:** Molecular docking binding energies.

Core Target	PDB ID	Binding Energy (kcal/mol)
PPARγ	1fm9	−8.4
MAPK3	4qtb	−9.0
ESR1	6psj	−8.9
PTGS2	5ikq	−8.9
HMGCR	1dqa	−7.9
PPARα	1i7g	−8.4

**Table 2:** Binding free energy and energy decomposition (kcal/mol).

Energe Component (Kcal/mol)	ESR1	HMGCR	MAPK3	PPARα	PPARγ	PTGS2
△VDWAALS	−46.11	−47.11	−25.03	−42.11	−28.30	−32.30
△EEL	135.77	−20.85	−38.48	95.81	−0.89	15.90
△EPB	−117.03	40.99	45.99	−77.88	15.64	−0.71
△ENPOLAR	−4.47	−4.12	−2.81	−4.02	−2.79	−3.05
△EDISPER	0.00	0.00	0.00	0.00	0.00	0.00
△GGAS	89.66	−67.95	−63.51	53.70	−29.19	−16.40
△GSOLV	−121.50	36.87	43.18	−81.90	12.85	−3.76
△TOTAL	−31.84	−31.08	−20.33	−28.20	−16.34	−20.16

**Table 3:** Tumor weight and tumor inhibition rate in BALB/c mice of each group.

Grouping	*n*	Weight	Tumor Inhibition Rate
Control	6	1.416 ± 0.081	-
OA	6	0.975 ± 0.039 *	31.16%
OA + GW6471	6	0.899 ± 0.062 *	40.04%

* Compared with the Control group, it is statistically significant (*p* < 0.05).

**Table 4:** Database Summary.

Database	URL
PubChem	https://pubchem.ncbi.nlm.nih.gov/ (accessed on 30 January 2025)
SwissTargetPrediction	https://swisstargetprediction.ch/ (accessed on 30 January 2025)
Genecards	https://www.genecards.org/ (accessed on 31 January 2025)
OMIM	https://www.omim.org/ (accessed on 31 January 2025)
Venny2.1.0	https://bioinfogp.cnb.csic.es/tools/venny/index.html (accessed on 31 January 2025)
STRING	https://cn.string-db.org/ (accessed on 1 February 2025)
DAVID	https://davidbioinformatics.nih.gov/ (accessed on 1 February 2025)
WeiShengXin	https://www.bioinformatics.com.cn/ (accessed on 1 February 2025)

**Table 5:** qRT-PCR primer sequences.

Gene	Primer	Sequence (5′–3′)	PCR Products/bp
*GAPDH*	Forward	GTGGACCTGACCTGCCGTCTAG	149 bp
	Reverse	GAGTGGGTGTCGCTGTTGAAGTC	
*PPARA*	Forward	CAAGCTGGTGTATGACAAGTGC	97 bp
	Reverse	TGTGACATCCCGACAGAAAGG	
*FASN*	Forward	CCTCAGCCGCCATCTACAACATC	117 bp
	Reverse	GCCAGCGTCTTCCACACTATGC	
*CPT1A*	Forward	CATGTCCAGCCAGACGAAGAACG	192 bp
	Reverse	CGACTCATCTTGCCGTGCTCAG	
*HMGCR*	Forward	GTGGCCCAGTTGTGCGTCTTC	94 bp
	Reverse	GCCTCCTTTATCACTGCGAACCC	

## Data Availability

The datasets generated and analyzed during this study are accessible through online repositories, with references provided within the main text and Supplementary Materials File S1.

## Supplementary Materials

The following supporting information can be downloaded at: https://www.mdpi.com/article/10.3390/ijms27104595/s1.

## Abbreviations

BP	Biological Process
CC	Cellular Component
CPT1A	Carnitine Palmitoyltransferase 1A
DMSO	Dimethyl Sulfoxide
ESR1	Estrogen Receptor 1
FASN	Fatty Acid Synthase
GO and KEGG	Gene Ontology and Kyoto Encyclopedia of Genes and Genomes
HCC	Hepatocellular Carcinoma
HMGCR	3-Hydroxy-3-Methylglutaryl-CoA Reductase
MAPK3	Mitogen-Activated Protein Kinase 3
MF	Molecular Function
OA	Oleanolic Acid
PPARα	Peroxisome Proliferator-Activated Receptor Alpha
PPAR	Peroxisome Proliferator-Activated Receptor
PPARβ	Peroxisome Proliferator-Activated Receptor Beta/Delta
PPARγ	Peroxisome Proliferator-Activated Receptor Gamma
PPI	Protein–Protein Interaction
PPREs	Peroxisome Proliferator Response Elements
PTGS2	Prostaglandin–Endoperoxide Synthase 2
RXRα, RXRβ, RXRγ	Retinoid X Receptor Alpha, Beta, Gamma
