# Supplementary material for: Integrating Network Pharmacology and Experimental Validation of Oleanolic Acid Targeting the PPARα-CPT1A Axis to Modulate Lipid Metabolism in Hepatocellular Carcinoma Cells
Source: Int J Mol Sci. 2026 May 20;27(10):4595. doi: 10.3390/ijms27104595 (PMC13207446; doi:10.3390/ijms27104595)
Supplement: Supplementary file 1 [file ijms-27-04595-s001.zip › Figures S1 and S2.pdf]

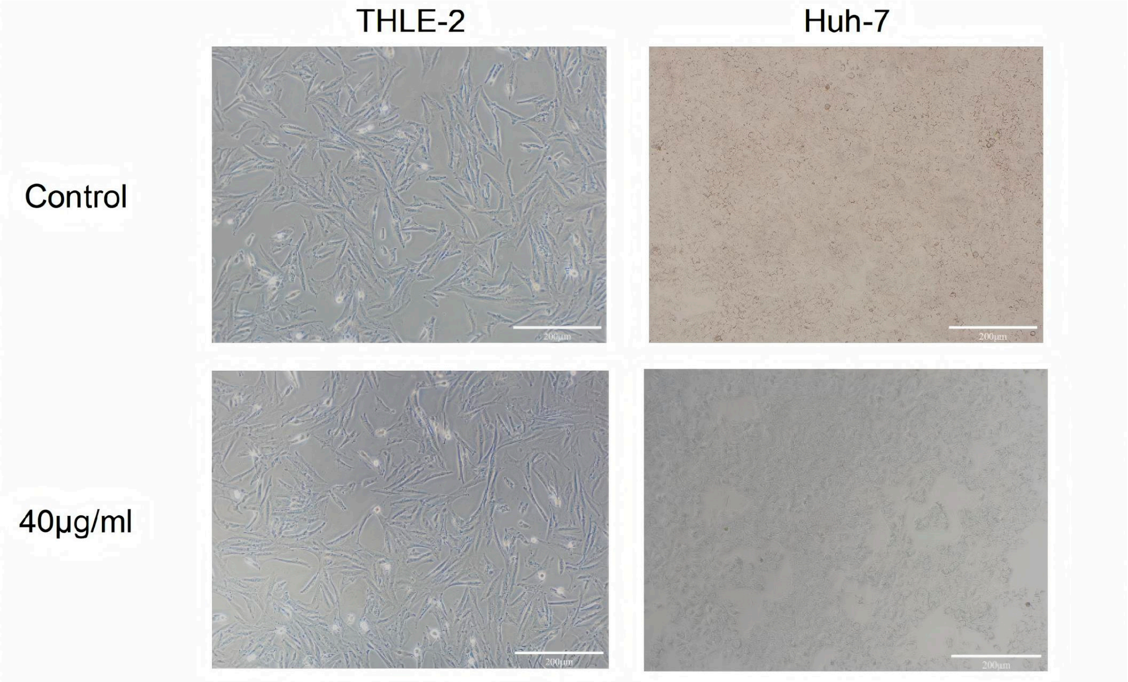

Figure S1. Comparison of cell morphology between Huh-7 and THLE-2 cells.

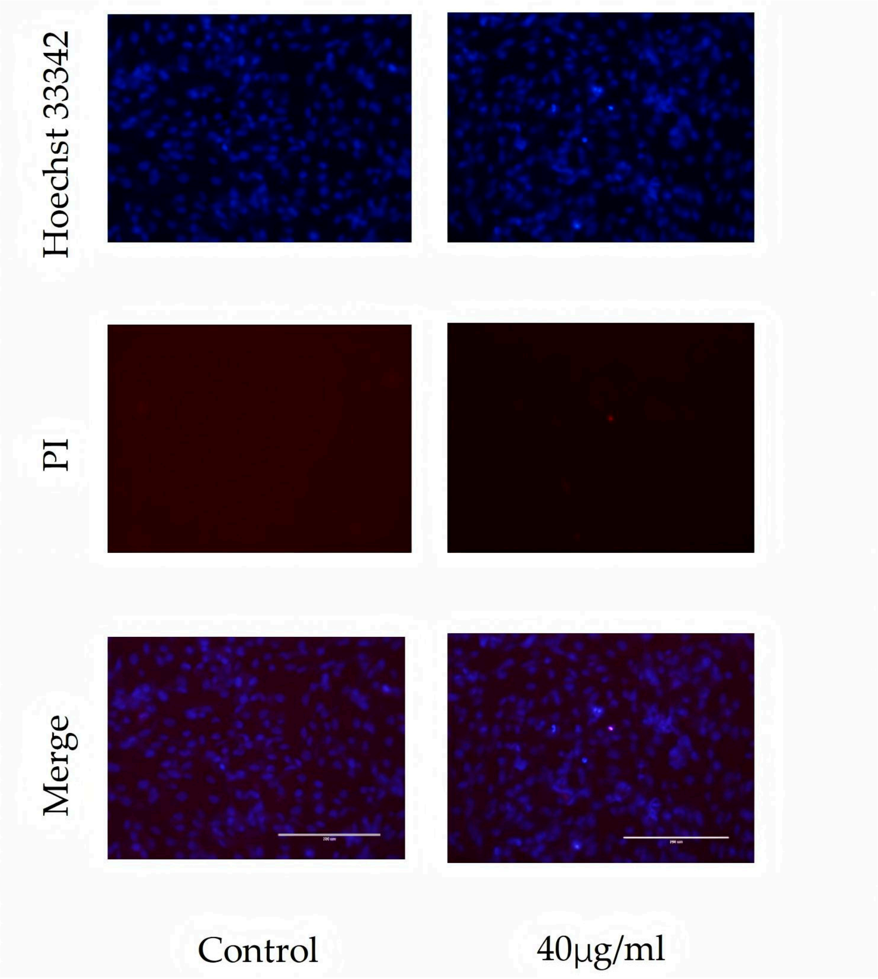

Figure S2. Hoechst 33342/PI Staining of THLE-2 cells.
